# Draft Genome Sequences of *Dysgonomonas* sp. Strains GY75 and GY617, Isolated from the Hindgut of Reticulitermes flavipes

**DOI:** 10.1128/MRA.00079-21

**Published:** 2021-03-25

**Authors:** Charles M. Bridges, Daniel J. Gage

**Affiliations:** a Department of Molecular and Cell Biology, University of Connecticut, Storrs, Connecticut, USA; Indiana University, Bloomington

## Abstract

*Dysgonomonas* species are facultative heterotrophs capable of growth on lignocellulose-derived polysaccharides. *Dysgonomonas* species harbor myriad genes involved in glycan modification and are well suited to the lignocellulose-rich conditions within the termite hindgut. Here, we report draft genome sequences for *Dysgonomonas* sp. strains GY75 and GY617, isolated from the hindgut of Reticulitermes flavipes.

## ANNOUNCEMENT

*Dysgonomonas* species are facultative anaerobic heterotrophs enriched in polysaccharide-replete environments and are an emerging source of potentially novel glycan-modifying genes. Here, we present draft genome sequences for *Dysgonomonas* sp. strains GY75 and GY617, isolated from the hindgut of the lower termite Reticulitermes flavipes.

The hindguts were extirpated (1) from worker termites collected in Granby, CT (2). The contents were serially diluted, plated onto peptone-yeast extract-blood glucose (PYBG) agar (2), and incubated for 5 days at 22°C under anaerobic conditions (89% N_2_, 5.5% H_2_, 5.5% CO_2_). Isolates were purified and maintained on PYBG under the same conditions. 16S rRNA gene sequences placed isolates GY75 and GY617 in the genus *Dysgonomonas* (2) (available under GenBank accession no. MT340879 and MT340880, respectively). Genomic DNA was prepared using an Epicentre MasterPure DNA and RNA purification kit. DNA was quantified using a Qubit fluorometer, and quality was determined by gel electrophoresis and with a NanoDrop spectrophotometer. The purified DNA was mechanically sheared, and 550-bp Illumina TruSeq PCR-free libraries were prepared. The libraries were sequenced on an Illumina MiSeq instrument using a 2 × 250-bp v2 kit. Three independently generated read sets were obtained for each isolate. The read sets were independently paired, contaminant filtered, adapter trimmed, quality trimmed, and filtered (Q20 threshold) using BBTools v38.86 (https://sourceforge.net/projects/bbmap). The read sets were analyzed with FastQC v0.11.9 (https://www.bioinformatics.babraham.ac.uk/projects/fastqc). The quality-filtered read sets were assembled with A5-miseq v20160825 (3) (default settings) on KBase (4) and using SPAdes v13.14.1 (5) (“--careful”). The assemblies were merged with MAC (19 July 2020) (6) (default settings) using concatenated, forward (or reverse) reads from all three read sets. The filtered, paired read sets were concatenated and mapped to MAC-merged assemblies with Bowtie 2 v2.4.1 (7) (“--very-sensitive-local”), using the unpaired (“-U”) and paired (“--interleaved”) options. SAMtools v1.10 (8) was used for conversion, sorting, and indexing the BAM files. The merged assemblies were iteratively polished seven times using Pilon v1.23 (9) (“--fix all,breaks,novel”), utilizing the paired (“--frags”) and unpaired (“--unpaired”) Bowtie 2-generated BAM files. The assembly statistics were calculated using QUAST v5.0.2 (10). Scaffolds were manually curated and submitted to the NCBI PGAP v4.13 server (11) for genome annotation. The genome sequences were screened for completeness and contamination using CheckM v1.0.18 (12) on KBase. The genome sequences were submitted to the Type Strain Genome Server (TYGS) (13) to calculate the whole-genome digital DNA-DNA hybridization (dDDH) values between cultured *Dysgonomonas* spp. (with a corresponding RefSeq genome sequence as of 10 November 2020). The average amino acid identity (AAI) between orthologous genes was computed using CompareM v0.1.2 (https://github.com/dparks1134/comparem). Carbohydrate-active enzyme (CAZy) domains were identified using the dbCAN2 (14) server, and polysaccharide utilization loci (PUL) were identified using PULpy (15) (17 September 2020).

*Dysgonomonas* isolate GY617 was closely related to *Dysgonomonas* sp. strain HGC4, which was independently isolated from a geographically distinct colony of *R. flavipes* (16). *Dysgonomonas* isolate GY75 harbored the largest genome sequence within the genus, clustered within the type species Dysgonomonas gadei using all metrics (Table 1), and similarly contained *nif* genes encoding nitrogenase.

**TABLE 1:** Genomic details of *Dysgonomonas* sp. strains GY75 and GY617

Parameter	Data for strain:	
	GY75	GY617
BioProject accession no.	PRJNA656570	PRJNA656570
BioSample accession no.	SAMN16421156	SAMN16421157
SRA accession no.	SRR12822925	SRR12822924
WGS accession no.	JADCLQ000000000.1	JADCLR000000000.1
RefSeq accession no.	GCF_015234155.1	GCF_015233815.1
Taxonomy ID no.^*a*^	2780419	2780420
		
No. of raw sequencing reads	2,873,620	4,137,364
No. of filtered reads	2,257,726	3,395,260
Mean scaffold coverage^*b*^ (×)	72	140
No. of scaffolds >100 bp	73	44
No. of contigs >100 bp	83	53
Largest scaffold size (bp)	891,650	893,182
Genome size (bp)	5,983,618	4,757,534
*N*_50_ (bp)	234,682	356,261
*L*_50_	5	4
G+C content (%)	40.02	35.92
		
No. of coding sequences	4,830	3,911
No. of protein-coding sequences	4,722	3,836
No. of tRNAs	43	43
Completeness (%)	99.45	100
Contamination (%)	2.19	0
		
Closest neighbor, 16S rRNA gene (% identity)	Dysgonomonas gadei ATCC BAA-286^*c*^ (100)	*Dysgonomonas* sp. strain HGC4^*d*^ (99.87)
Closest neighbor, whole genome (% dDDH^*e*^)	*D. gadei* ATCC BAA-286^*c*^ (85.8)	*Dysgonomonas* sp. strain HGC4^*d*^ (87.1)
Closest neighbor, protein-coding sequences	*D. gadei* ATCC BAA-286^*c*^	*Dysgonomonas* sp. strain HGC4^*d*^
Orthologous fraction of genes^*f*^ (% of total)	86.16	88.04
AAI between orthologs^*g*^ (%)	98.05	98.27
		
Total no. of CAZy domains^*h*^	308	274
No. of glycosyl hydrolase domains	235	190
No. of carbohydrate esterase domains	11	11
No. of polysaccharide lyase domains	5	9
No. of carbohydrate-binding module domains	18	11
No. of glycosyl transferase domains	39	53
No. of protein-coding sequences with ≥1 CAZy domain (% of total)	291 (6.16)	259 (6.75)
Total no. of PULs	67	40
No. of CAZy-associated PULs (% of total)	45 (67.2)	31 (77.5)

a ID, identifier.

b Determined using unpaired filtered reads mapped to Pilon-polished assembly using Bowtie 2.

c RefSeq assembly accession no. GCF_000213555.1.

d RefSeq assembly accession no. GCF_014840235.1.

e Digital DNA-DNA hybridization values were calculated using TYGS formula d_4_ (sum of all identities found in high-scoring pairs [HSPs] divided by the overall HSP length).

f The number of orthologs divided by the minimum number of protein-coding genes from either genome.

g Average amino acid identity between orthologous fraction of genes.

h Domains identified by HMMER and at least one other tool (HotPep or DIAMOND).

### Data availability.

The genome assemblies for *Dysgonomonas* isolates GY75 and GY617 were submitted to NCBI under BioProject accession no. PRJNA656570 and whole-genome shotgun sequence (WGS) accession no. JADCLQ000000000.1 and JADCLR000000000.1, respectively. Detailed information can be found in Table 1.
